# Kojic Acid Promotes Apoptosis-like Death and Cell Cycle Arrest in *Leishmania* (*Leishmania*) *amazonensis* Promastigotes

**DOI:** 10.1021/acsomega.5c04741

**Published:** 2025-08-11

**Authors:** Danny B. Ventura Silva, Adan J. Galué-Parra, Poliana Queiroz-Souza, Luiz P. C. Pinheiro, Vinicius Pacheco, Chubert B.C. de Sena, Jose L. M. do Nascimento, Edilene O. Silva

**Affiliations:** † Laboratory of Structural Biology, Institute of Biological Sciences, 37871 Federal University of Pará, Belém, Pará 66075-110, Brazil; ‡ Postgraduate Program in Neuroscience and Cell Biology, Federal University of Pará, Belém, Pará 66075-110, Brazil; § Laboratory of Molecular and Cellular Neurochemistry, Institute of Biological Sciences, Federal University of Pará, Belém Pará 66075-110, Brazil; ∥ National Institute of Science and Technology in Neuroimmunomodulation (INCT-NIM), Rio de Janeiro 21040-900, Brazil; ⊥ National Institute of Science and Technology in Structural Biology and Bioimaging (INCT-INBEB), Rio de Janeiro 21941-902, Brazil

## Abstract

Leishmaniasis is a group of neglected tropical diseases caused by protozoa of the genus Leishmania that are present in about 90 countries. More than 20 species are responsible for the infection, causing varying clinical manifestations. Leishmaniasis treatment includes pentavalent antimonials that have been used for decades as the first-choice drug. However, due to their severe side effects, high cost, and protozoan resistance, finding new, affordable, and safe drug alternatives for leishmaniasis treatment is necessary. Kojic acid (KA) promotes leishmanicidal activity for both promastigotes and amastigotes, in vitro and in vivo. The objective of this study was to evaluate the mechanism of action of KA on promastigotes of *Leishmania (Leishmania) amazonensis*. Our findings demonstrate that KA induces direct leishmanicidal effects by promoting apoptosis-like cell death, oxidative stress, ultrastructural changes, and cell cycle disruption in *Leishmania (L.) amazonensis* promastigotes. These results position KA as a promising candidate for future antileishmanial drug development.

## Graphical abstract

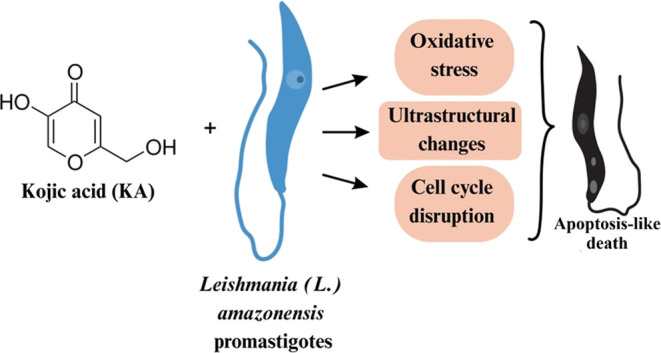

## 1 Introduction

Leishmaniasis is a group of neglected tropical diseases caused by protozoa of the genus *Leishmania*, which are present in more than 90 countries. According to the World Health Organization, it is estimated that 700 thousand to 1 million new cases occur annually, representing a significant public health concern.^1–3^ Leishmaniasis is transmitted by sandflies of the genus *Lutzomyia* or *Phlebotomus* through a digenetic life cycle that has the evolutionary forms of promastigotes and amastigotes.^1,4–6^

Leishmania promastigotes are extracellular and flagellated-motile forms that are inoculated into the host upon blood shedding by the sandfly vector. After the inoculation, neutrophils and macrophages phagocytose the promastigotes, which are later transformed into intracellular amastigotes, ultimately establishing the infection by residing in the interior of mammalian macrophages.^7,8^

Leishmaniasis presents three primary clinical forms: cutaneous leishmaniasis (CL), mucocutaneous leishmaniasis (MCL), and visceral leishmaniasis (VL), the latter of which is almost invariably fatal without treatment. Currently, the available treatment options are limited and only moderately effective.^9^ Pentavalent antimonials, amphotericin B, and pentamidines have been used in therapeutic management for decades. However, these drugs incur high toxicity and severe side effects to the patient, along with high cost and parasitic resistance.^10–12^ Therefore, there is a need to seek safer and more cost-effective alternatives to potentially replace current approaches as new therapeutic methods.

Kojic acid (KA), a metabolite produced by *Aspergillus* *spp*. and *Penicillium* *spp.*, demonstrates protozoan activity, as well as antimicrobial, antiviral, anti-inflammatory, antitumor, antidiabetic, radioprotective, macrophage-activating, monocyte differentiation, and metal-chelating activities for iron, copper, zinc, and magnesium.^13–18^ Previous studies by our research group indicate that KA exerts effects against Leishmania by increasing ROS, producing intense intracellular vacuolation, promoting vesicular body formation in the flagellar pocket, increasing lipid-like bodies, and causing mitochondrial swelling in intracellular amastigotes.^16^ However, its effects on the promastigote form of Leishmania are not well understood and warrant further investigation. Furthermore, it is not clear whether KA induces apoptosis, necrosis, or other types of cell death in the promastigote form. Therefore, the aim of this study was to explore the role of KA in the cell death mechanism of Leishmania promastigotes. Understanding this relationship could provide valuable insights into the biology of the parasite and help in the search for new treatments.

## 2 Materials and Methods

### 2.1 Obtaining and Using Kojic Acid (KA)

KA was purchased from Sigma and diluted to 1 mg/mL in the Roswell Park Memorial Institute (RPMI) medium. The concentration of 50 μg/mL was selected based on previous dose–response studies conducted by our group using non-nanoformulated KA, which showed this concentration to be effective in inducing leishmanicidal activity.^16^ This study specifically investigates the mechanism of the free compound to build a foundational understanding, complementing our recent research on more complex nanoformulations.^19^

### 2.2 Parasites

*Leishmania amazonensis* promastigotes (MHOM/BR/2361) were obtained from Instituto Evandro Chagas in the NNN medium. Subsequently, they were maintained in RPMI medium supplemented with 10% fetal bovine serum (FBS) in a BOD (biochemical oxygen demand) incubator at 27 °C. The promastigote forms were used on the fourth day of cultivation.

### 2.3 Morphological Analysis of *Leishmania* (*L.*) *Amazonensis* Promastigotes for Scanning Electron Microscopy

The promastigotes were treated with 50 μg/mL KA for 24 h. Subsequently, the promastigotes were washed twice with PBS and fixed for 1 h in a solution containing glutaraldehyde (2.5%) and cacodylate buffer (0.1 M). At the end of the incubation, they were fixed on coverslips with poly-l-lysine at room temperature for 40 min for adhesion. Subsequently, 3 washes were performed in cacodylate buffer (0.1 M) and dehydrated in an increasing series of ethanol (30–100%) for 10 min for each series at room temperature. After dehydration, the coverslips were transferred to the critical point apparatus, placed in appropriate supports for metallization, and analyzed using a VEGA TESCAN 3 SEM instrument.

### 2.4 Detection of Reactive Oxygen Species

The promastigotes were treated with 50 μg/mL KA for 24 h. Afterward, they were removed from the wells and centrifuged to enable reactive oxygen species (ROS) detection using the CellRox Green Kit (Flow Cytometry Assay-Invitrogen). For this, the parasites were washed with PBS pH 7.2 and incubated for 45 min at 27 °C with the fluorescent marker (CellRox) at a concentration of 5 μM, previously diluted in PBS. At the end of the incubation, the promastigotes were washed and resuspended in PBS for analysis using a BD FACSCanto II flow cytometer. Promastigotes incubated with 5 μL of miltefosine were used as a positive control.

### 2.5 Cell Death Analysis

Promastigotes (1 × 10^6^ cells/ml) were treated with 50 μg/mL KA for 24 h. Subsequently, they were incubated with 5 μL of annexin V-FITC (Invitrogen) in binding buffer for 15 min at room temperature. Then, 500 μL of the inoculum was placed in cytometry tubes before adding 50 μL of propidium iodide (PI) and analyzing using a BD FACSCanto II flow cytometer. Untreated promastigotes were used as negative controls, and parasites incubated with miltefosine (40 μM) for 24 h were considered positive controls.

### 2.6 Quantification of Lipid Bodies by Flow Cytometry Using Bodipy 493/503

For quantification of LBs in each sample, promastigotes treated with 50 μg/mL KA for 24 h were washed in PBS, pH 7.2, and incubated with Bodipy 493/503 (Invitrogen) at a concentration of 10 μg/mL in PBS, followed by reading on a BD FACSCanto II flow cytometer. Promastigotes incubated with 20% FBS were used as a positive control.

### 2.7 Determination of the Cell Cycle

Promastigotes (1 × 10^6^ cells/mL) treated with 50 μg/mL KA for 24 h were washed twice with PBS, resuspended, and incubated for 30 min in a 70% methanol solution in ice-cold PBS and fixed using the same solution for 24 h at 4 °C. Subsequently, the parasites were washed twice with PBS, resuspended, and incubated in a solution containing 485 μL of PBS, 5 μL of PI, and 10 μL of RNase (10 μg/mL) for 45 min at 37 °C. Cells were analyzed using a BD FACSCanto II flow cytometer. As a positive control, parasites were incubated with miltefosine (40 μM).

### 2.8 Statistical Analysis

Tests were carried out in triplicate, and the data obtained were analyzed with the GraphPad Prism software, using ANOVA with Tukey and Dunnett post-tests and a significance level of *p* < 0.05.

## 3 Results

To verify whether the treatment induced ultrastructural changes in the external morphology of the protozoan, SEM analysis was performed. The untreated control group presented typical morphology (Figure 1A). The group treated with 50 μg/mL KA displayed significant morphological changes, such as cellular rounding, duplication, and shortening of the flagellum (Figure 1B,C).

**Figure 1:**
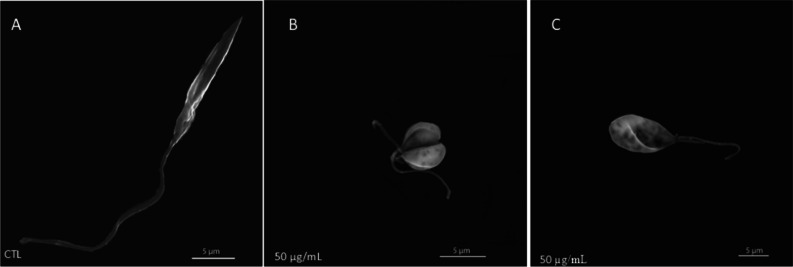
Ultrastructural analysis by SEM of the promastigote form of *L.* (*L.*) *amazonensis*, treated or not with KA. (A) Untreated group; promastigotes showed typical morphology. (B,C)­Promastigotes treated with 50 μg/mL KA present flagellar duplication, a shortened body, and decreased cell volume. Scale bar, 5 μm.

The effect of KA on the generation of oxidative stress in the protozoan was also investigated using the CellROX Green probe. An increase in ROS production was observed after 24 h of treatment with 50 μg/mL KA, compared to the control group (Figure 2). Miltefosine (40 μM), which induced a significant increase in ROS in promastigote forms, was used as a positive control for the experiment.

**Figure 2:**
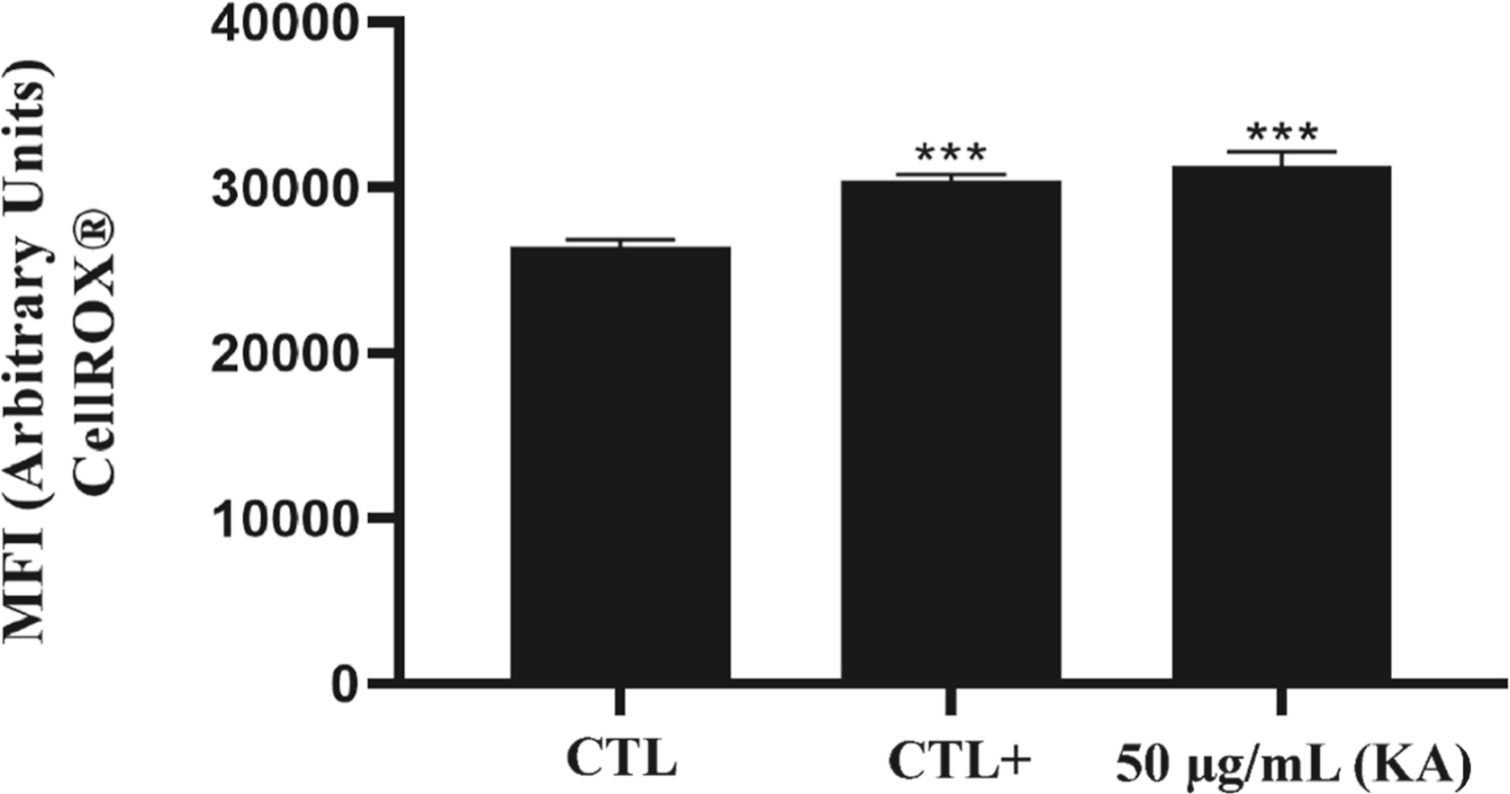
Oxidative stress generation in *L.* *(L.)* *amazonensis* promastigotes treated with 50 μg/mL KA. CellRox and flow cytometry were used to assess ROS. Miltefosine (40 μM) was used as a positive control. ***p < 0.001.

Annexin V-FITC (Invitrogen) binding was used to verify whether KA caused organism death. KA at a concentration of 50 μg/mL induced approximately 70.9% apoptosis-like cell death (Figure 3C), compared with the control group, which exhibited approximately 97.66% viable promastigotes (Figure 3A). The positive control group treated with miltefosine induced apoptosis-like cell death in approximately 45.31% of cells (Figure 3B). Figure 3D summarizes the distribution of promastigote death as a percentage across the experimental groups.

**Figure 3:**
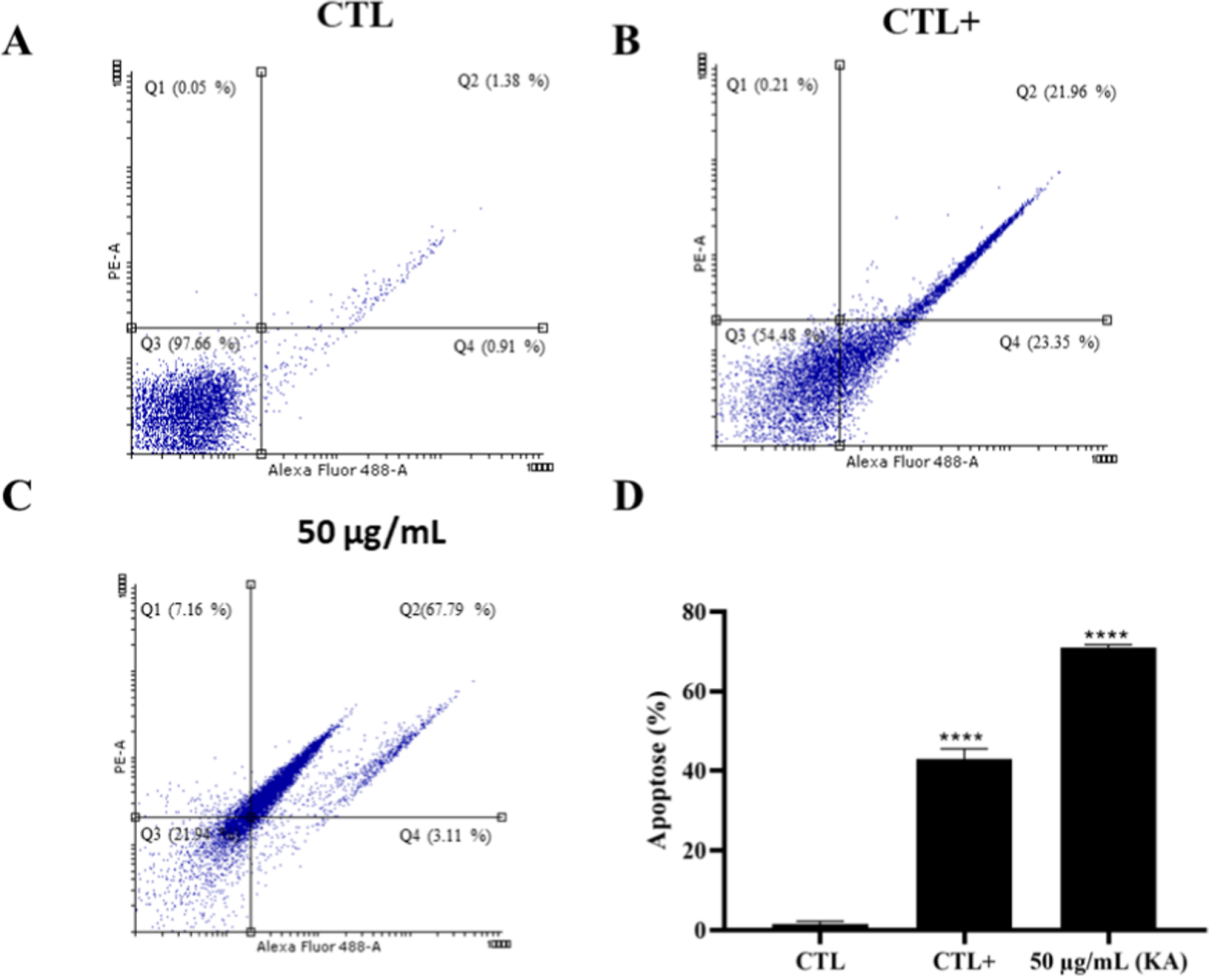
Analysis of cell death in promastigotes of *L. (L.) amazonensis* treated with KA by flow cytometry. A—untreated control; B—miltefosine (40 μM) positive control; (C)—promastigotes treated with 50 μg/mL KA; (D)—apoptosis-like cell death rates for experimental groups. ****p < 0.0001.

Interestingly, treatment with KA induced an increase in the amount of lipid bodies in promastigotes (Figure 4), compared to the untreated group.

**Figure 4:**
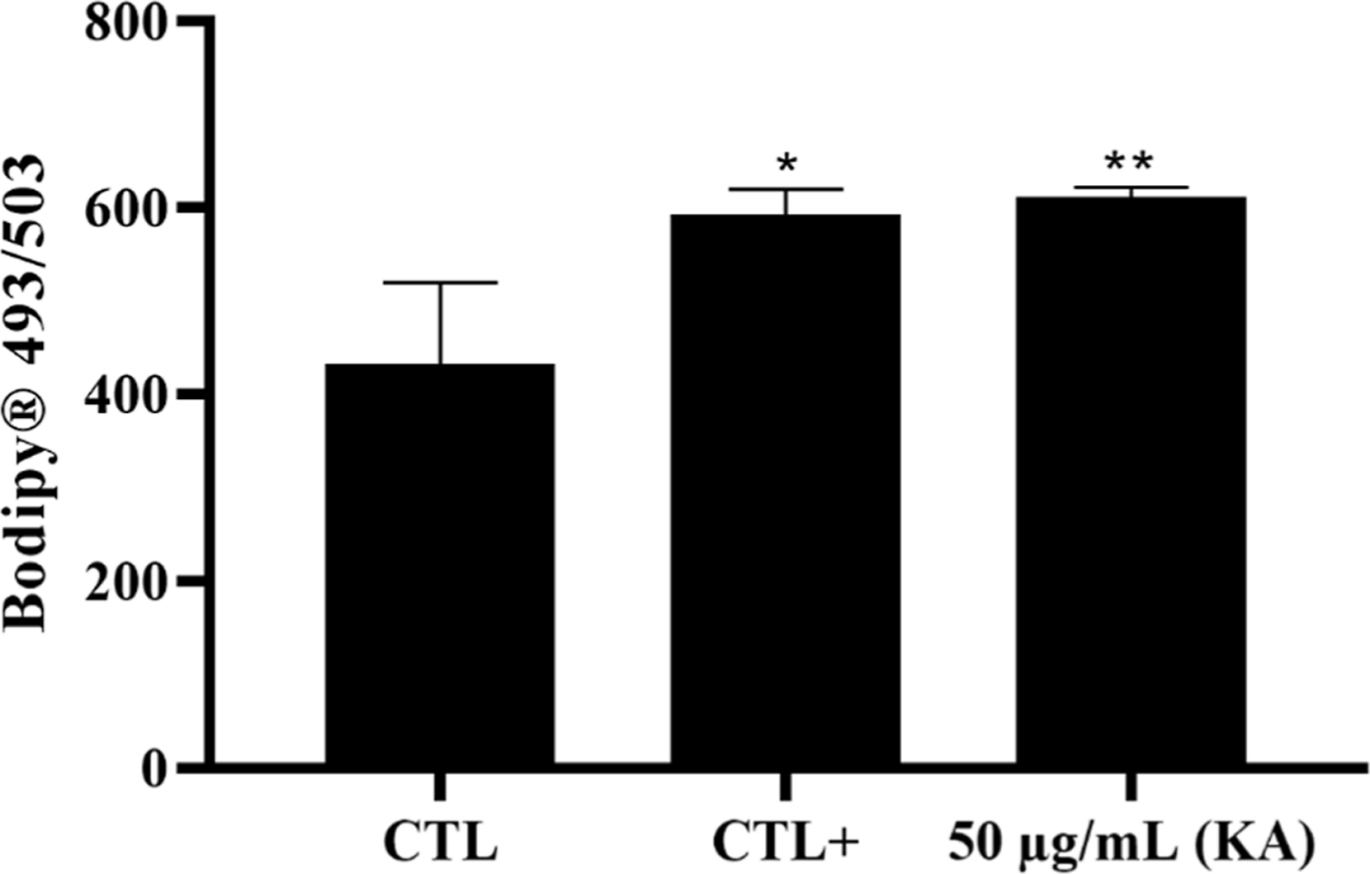
Quantification of lipid bodies in promastigotes of *L.* (*L.*) *amazonensis* treated with KA by flow cytometry. Note the increase in lipid bodies at the concentration of 50 μg/mL KA. FBS (20%) was used as a positive control. (*) *p* < 0.05 significant; (**) *p* < 0.01.

To evaluate possible changes during the cell division process, promastigotes were treated with 50 μg/mL KA. This treatment resulted in DNA fragmentation, accompanied by a significant increase in the proportion of promastigotes in the sub-G0/G1 phase (31.24%), compared to the untreated control (5.04%). Additionally, KA treatment induced cell cycle arrest, as demonstrated by a reduction in the percentage of promastigotes in the G2-M phase (8.01%) compared to the untreated control (20.12%) (Table 1). These findings are also depicted in Figure 5.

**Table 1:** Percentages of *L.* (*L.*) *amazonensis* Treated with KA in Different Cell Cycle Phases

cell cycle phases	CTL (%)	50 μg/mL KA (%)
sub G_0_/G_1_	5.04 ± 0.54	31.24 ± 7.93
G_0_/G_1_	59.70 ± 0.36	49.89 ± 4.65
S	10.38 ± 0.63	9.55 ± 0.87
G_2_/M	20.12 ± 0.47	8.01 ± 2.22

**Figure 5:**
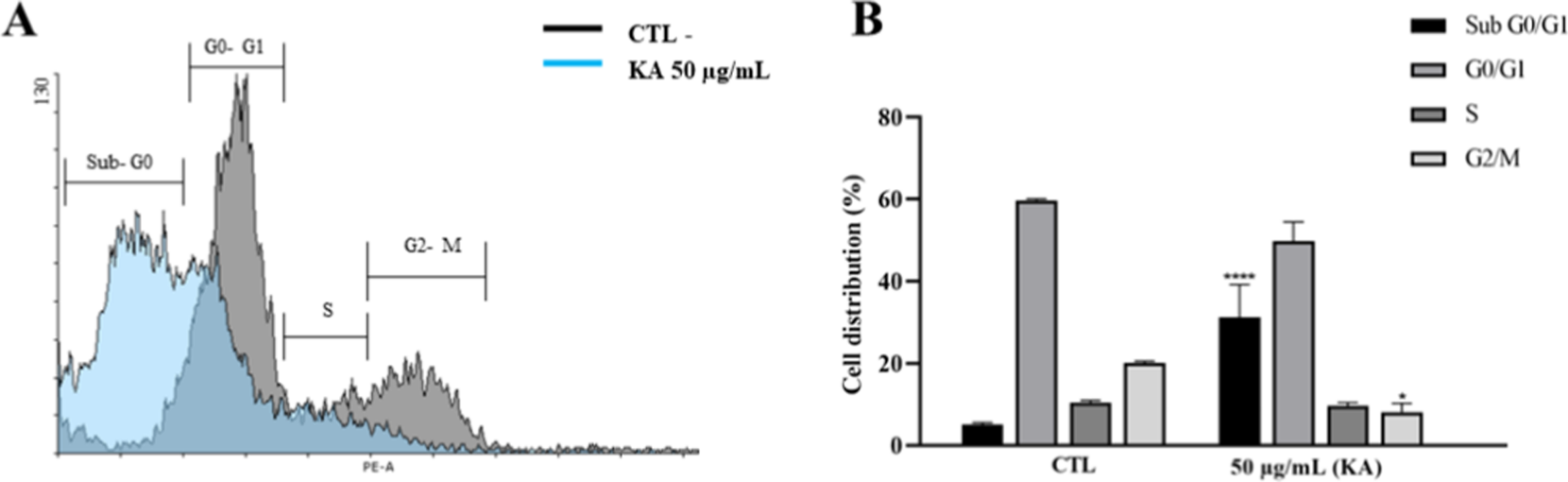
Cell cycle analysis of *L. (L.) amazonensis* promastigotes treated with KA by flow cytometry. (A)—Histogram showing the control group (gray) and promastigotes treated with 50 μg/mL KA (blue); (B)—cell cycle distribution analysis demonstrating arrest at the sub-G0/G1 and G2/S phases after 50 μg/mL KA treatment. Statistical analysis was performed using ANOVA, followed by the Bonferroni posthoc test. (*) *p* < 0.05, considered statistically significant; (****) *p* < 0.0001, considered highly significant.

## 4 Discussion

Leishmaniasis is a group of neglected tropical diseases with limited treatment options, many of which are toxic, expensive, and only moderately effective.^10–12^ In this study, we evaluated the in vitro leishmanicidal effect of KA, a potential antileishmanial candidate, in promastigotes of *L.* (*L.*)­*amazonensis*. We found that KA triggers an apoptosis-like response in these promastigotes, altering the parasite’s morphology and modulating its cell cycle.

Our ultrastructural analysis using SEM adds to previous findings by focusing on the external morphological effects of non-nanoformulated KA. While our earlier work using transmission electron microscopy demonstrated that a nanoemulsion of KA caused internal organelle damage,^19^ this study reveals that free KA affects the surface of the parasite. We observed significant changes including flagellar shortening or duplication, cellular rounding, and reduced cell volume. Although these morphological changes were not measured, they were consistently observed across different fields and replicates.

These qualitative changes, including flagellar duplication and cell rounding, suggest a robust phenotypic response to the KA treatment. These alterations to the parasite’s surface and cytoskeleton are critical, as they would directly impair promastigote motility and its ability to initiate host cell invasion.

Earlier studies have demonstrated that KA induces oxidative stress in parasitic cells.^13,19,20^ We observed an increase in ROS production in promastigotes following KA treatment, suggesting that oxidative stress contributes to their leishmanicidal activity.

Leishmania parasites maintain the balance of their redox state. However, if reactive oxygen species (ROS) increase over a prolonged period, even if this does not cause immediate cell damage, the balance is lost and antioxidants are disturbed in favour of oxidants, such as the trypanothione reductase pathway.^21^ This sustained oxidative stress may activate downstream signalling pathways, resulting in mitochondrial damage and ultimately cell death.

This mechanism is consistent with that of clinically approved antileishmanial agents, such as miltefosine and amphotericin B.^22–24^ The oxidative imbalance triggered by KA may impair mitochondrial function, leading to apoptosis-like response, as supported by the detection of Annexin V binding, a marker of apoptosis, by flow cytometry.^25^

Oxidative stress is recognized as an important mechanism for parasite clearance.^26^ To counteract this, *Leishmania* uses antioxidant systems, such as trypanothione reductase and superoxide dismutase (SOD), to defend against the host’s immune response.^21^ The accumulation of KA-induced ROS generation may have led to mitochondrial damage and phosphatidylserine externalization, which is a hallmark of apoptosis. Similar processes have been reported with other leishmanicidal agents, including amphotericin B, which has been observed to cause apoptosis mediated by ROS.^27^

Leishmania species do not possess the typical caspase enzymes responsible for executing apoptosis in metazoans; therefore, the process they undergo is more accurately termed “apoptosis-like cell death” (ALCD). This process, however, shares key morphological and biochemical hallmarks with classical apoptosis, including phosphatidylserine externalization, as detected by Annexin V binding, mitochondrial dysfunction, and DNA fragmentation.^25,28^ The execution of ALCD in Leishmania is thought to be mediated by other proteases, such as metacaspases and calpains.^29^

The accumulation of lipid bodies suggests that KA disrupts lipid metabolism, which is essential for membrane synthesis, signal transduction, and energy homeostasis.^30,31^ Metabolic and lipid disruptions are associated with the increase of lipid body formation and ROS production in leishmaniasis.^32,33^ During in vivo infection, an increase in lipid bodies may be associated with macrophage activation, as lipid accumulation is a common feature of immune cell responses to infection and oxidative stress.^34^

The cell cycle of *Leishmania* promastigotes is tightly regulated to ensure adequate replication and growth. Flow cytometric analysis revealed that the KA-treated group exhibited a significant accumulation of promastigotes in the sub-G0/G1 phase, which is indicative of DNA fragmentation and apoptosis.^28^ Moreover, KA treatment led to a notable reduction in the G2/M phase population, suggesting cell cycle arrest and the subsequent inhibition of parasite replication. Similar effects have been observed with artemisinin-derived essential oils, which also induce DNA damage and disrupt cell cycle arrest in *Leishmania*.^35,36^ These similarities reinforce the hypothesis that KA interferes with key regulatory pathways of the cell cycle, thereby impairing *Leishmania* proliferation.

As such, given its low toxicity, broad spectrum bioactivity, and low cost, KA may represent a promising antileishmanial agent.^15,16,17^ Future research should be directed toward assessing the in vivo efficacy of KA nanoformulations for both topical and systemic treatment. Previous studies have demonstrated that loading KA into nanoparticles, or combining it with bioproducts, can enhance its bioavailability and target specificity.^19,37^ Therefore, these strategies could significantly increase the therapeutic potential of KA against cutaneous leishmaniasis.

## 5 Conclusion

We, herein, demonstrate that KA directly induces apoptosis-like cell death in *L. (L.) amazonensis* promastigotes, mediated by oxidative stress, lipid body accumulation, and cell cycle arrest. Based on these findings, we hypothesize that similar mechanisms may be triggered in the amastigote form during active infection following KA treatment. These results highlight the potential of KA as a promising alternative to antileishmanial therapy.

## Abbreviations

KA: kojic acid
ROS: reactive oxygen species
LBs: lipid bodies
SEM: scanning electron microscopy
